# Anterior knee pain post-multiple surgeries for tibia fracture effectively managed with infrapatellar fat pad injection: a case report

**DOI:** 10.1186/s40981-022-00573-w

**Published:** 2022-10-10

**Authors:** Shinju Obara, Rieko Oishi, Yuko Nakano, Shin Kurosawa, Satoki Inoue

**Affiliations:** 1 Department of Anesthesiology, Fukushima Medical University, 1 Hikarigaoka, Fukushima, Fukushima 960-1295 Japan; 2 Center for Pain Management, Fukushima Medical University Hospital, 1 Hikarigaoka, Fukushima, Fukushima 960-1295 Japan

**Keywords:** Anterior knee pain, Infrapatellar fat pad, Pain clinic, Hydrorelease

## Abstract

**Background:**

The anterior knee compartment is filled by the infrapatellar fat pad (IFP) and has been emphasized as a source of anterior knee pain (AKP).

**Case presentation:**

A 51-year-old woman sustained a right tibial plateau and open diaphyseal fracture 2 years earlier. She remained with chronic AKP after undergoing bone fixations. Increased anterior portion of the IFP brightness and decreased adipose tissue gliding with flexion and extension compared to the unaffected side was shown on ultrasonography. An injection of 0.2% lidocaine between the patellar tendon and IFP, and into the IFP under ultrasound guidance, immediately relieved the pain. The pain kept recurring although injections were effective for a while; thus, surgery was scheduled. Scar tissue on the IFP surface was endoscopically excised and her pain dramatically reduced.

**Conclusion:**

This is the first report in which the detection of increased brightness on ultrasound of IFP and the injections into the IFP triggered an additional surgical intervention. Ultrasound evaluation and injection may be beneficial in pain clinic patients presenting with AKP and may provide an opportunity for diagnosis.

## Introduction

Anterior knee pain (AKP) can sometimes be challenging to control and is a relatively common chronic pain of the extremities. An intracapsular, extrasynovial structure that fills the anterior knee compartment, the infrapatellar fat pad (IFP) has been emphasized as a source of AKP although often overlooked or little considered until recently [1, 2]. Here, we describe a case of chronic AKP after tibial plateau fracture and metaphyseal release fracture repair, where ultrasound-guided IFP injections in an outpatient pain clinic setting was effective and of diagnostic significance in preparation for surgery.

## Case presentation

**Fig. 1:**
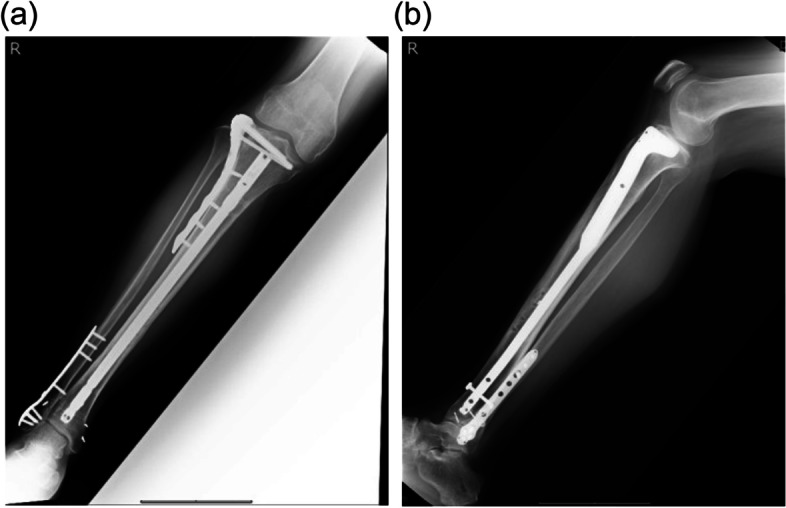
**a** Frontal and **b** lateral X-rays of the right tibia after bone fixations

The patient provided written informed consent for case report publication. A 51-year-old woman (body weight, 50 kg; height, 157 cm) sustained a right tibial plateau fracture and open diaphyseal fracture in a collision accident 2 years earlier. She underwent external and internal bone fixations at a different hospital, but remained with chronic AKP. Complete pain elimination after additional partial meniscectomy and pes anserinus debridement was not achieved (Fig. 1). Side effects of nausea and dizziness made it difficult for her to continue with duloxetine, tramadol, and loxoprofen. Intra-articular knee injections were also ineffective. Knee joint replacement was the sole surgical intervention considered. She did not wish to undergo the operation at that time and was referred to our pain clinic for pain relief. She refused an epidural block for pain management.

**Fig. 2:**
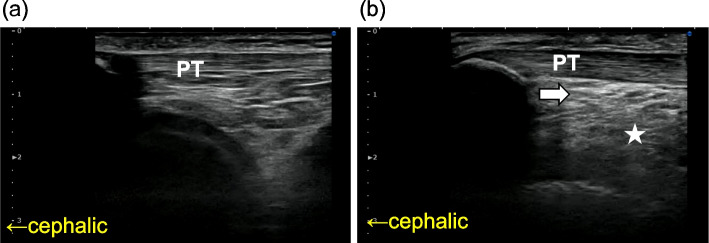
Ultrasound long-axis view of the unaffected (**a**) and affected (**b**) knee. White arrow indicates the infrapatellar fat pad with increased brightness; asterisk indicates slow-moving parts of the infrapatellar fat pad in the right knee. PT patella tendon

**Fig. 3:**
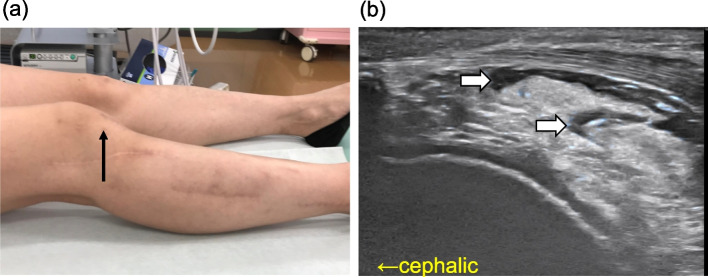
Injection into the infrapatellar fat pad. **a** Injection site (black arrow). The needle entry point is a 3-cm lateral from the center of the patellar tendon. **b** Ultrasound image after injection of 0.2% lidocaine 3 ml into each of the infrapatellar fat pad and the space between the patellar tendon and infrapatellar fat pad. Echo-free spaces (white arrow) are formed by local anesthetics

The patient presented with spontaneous posterior right patellar pain (visual analog scale, 60 mm), severe pain on impact at the start of walking, and difficulty in extension (30°). No local redness, swelling, or inflammatory findings on blood examination was noted. She complained of insomnia due to pain. Right quadricep atrophy was observed. Abnormal patellar deviation during flexion and extension of the knee joint was not observed. The Hoffa test (i.e., the test for assessing IFP tenderness; First, the firm pressure is applied to the IFP with the knee flexed; Then, the knee is fully extended, and increased pain indicates a positive) [2] was impossible to determine because the patient complained of maximum pain across the knee even when not fully performing the extension. Ultrasonography showed increased brightness of the anterior portion of the IFP (Fig. 2) and decreased adipose tissue gliding with flexion and extension compared to the unaffected side. A 3 ml of 0.2% lidocaine was injected using a 25G 38-mm needle (Fig. 3) between the patellar tendon and IFP and into the IFP under ultrasound guidance considering that the IFP lesion caused the pain, including its diagnostic significance, which immediately relieved the pain and allowed full knee extension (0°). Her insomnia improved as the first injection was enormously effective for 3 days. Injections were continued every 2 or 3 weeks for 9 months while tramadol 50 mg/day and prochlorperazine 10 mg/day was taken. Pain at rest was VAS 0 mm during 7–10 days after the injections. Pain at the initiation of walking was also reduced. The duration of effect was prolonged to about 14 days when 1.65 mg of dexamethasone was added to the drug solution. The patient did not complain of severe pain compared to the initial visit. No local infection or other complications associated with the injections were observed.

IFP was strongly considered to be the pain source based on the clinical course. The pain kept recurring, although injections were initially effective. Surgery was scheduled. Mild atrophy and fibrosis of the IFP was seen on preoperative MRI for definitive diagnosis. Surgery was performed under general anesthesia (propofol, remifentanil) and femoral nerve block. IFP impingement was not endoscopically observed during manual knee flexion–extension. Scar tissue was endoscopically excised on the IFP surface. The patient underwent rehabilitation for 2 weeks and was discharged without any trouble. Her pain was reduced to about 60% of its preoperative level without tramadol.

## Discussion

The IFP or Hoffa’s fat pad is an intracapsular, extrasynovial structure that is richly vascularized and innervated, and fills the anterior knee compartment [2]. The IFP may serve to absorb compressive stress by accommodating the dynamic shape and volume of the joint space in flexion extension [3], although the exact evolutionary purpose of the structure has not been elucidated [2]. The IFP is richly innervated by a branch of the posterior tibial nerve, and the posterior articular nerve [4]. The IFP is a pain-sensitive area [5], and its lesions have been suggested to be the cause of spontaneous or exercise pain in the knee [6]. The IFP is also involved in knee tenderness. With knee extension, the IFP is becomes captured between the femoral condyles and the tibial plateau [7], leading to injury, hypertrophy, and inflammation [8]. With the overload or trauma to the IFP or secondary to other knee disorders (e.g., meniscus injury [9], osteoarthritis [10], knee surgery [9], etc.) can cause inflammation, fatty tissue proliferation, and fibrosis, resulting in loss of flexibility, mobility impairment, and pain (i.e., Hoffa’s disease) [2, 9, 11].

At the time of the initial visit, the patient complained of pain at rest and during knee extension, typically caused by osteoarthritis or meniscus injuries, which was excluded because she had already undergone orthopedic fracture repair and meniscectomy. We assumed that the IFP lesion was the cause of the pain and performed a remarkably effective injection. The diagnosis of IFP lesions is usually made by MRI; fibrosis appears as a hypointense signal on T1- and T2-weighted images [2]. We have not found any reports of detecting increased brightness on ultrasound of IFP and using it as a diagnostic aid as far as we have explored.

Ultrasound observation showed that the IFP is deformed by flexion and extension of the knee joint [12]. Anterior and posterior movement speed of the very deep part of the IFP (Fig. 2) into the space between the femur and tibia seemed slower on the affected side than on the healthy side in extension and flexion, respectively. The significance regarding pain dynamics of the very deeper part IFP is unclear although the shallower region of the IFP has been shown to move forward with knee extension [13], which warrants future investigation.

AKP caused by IFP lesions is commonly initially treated with oral NSAIDs and conservative treatment (e.g., muscle training, taping, stretching) [3]. The effectiveness of injections has been considered a limited success [3], although effective cases have been reported [14]. Surgery is also considered in recalcitrant cases or cases in the presence of cartilaginous or bone nodules [1, 3]. Treatment options were limited in our case because the patient refused an epidural block at the first visit, and many oral chronic pain medications were difficult to continue due to side effects.

The injection into the IFP had a much longer duration of effect than the pharmacologic effect time of lidocaine (approximately 1 h). Other mechanisms of simple local anesthetic effects may have contributed to this in our case. Machida et al. [15] reported the case of a patient with superficial IFP scarring and sliding defect on ultrasound imaging after arthroscopic knee surgery, in whom hydrorelease with saline was effective. Hydrorelease is an injection using saline at the site with hyperechoic changes in the soft tissues [16]. Although the analgesic mechanism of hydrorelease is not well understood, the following mechanisms have been postulated: (1) washout of the various algesic substances in the interfascial space, (2) decreasing of the viscosity of the interfascial fluid, and (3) separation of the myofascial layers, which reduces muscular friction resulting in smooth movement [15, 16]. Fibrosis and local adhesions of IFP caused by the post-injury processes may have contributed to her anterior knee pain, and the temporary release of these adhesions by local anesthetics may have contributed to pain reduction in our case. Furthermore, there are reports that IFP pain is associated with neurogenic inflammation of the IFP with nociceptive nerve fibers containing substance P neurons [17]. Cases have been reported in which steroids may have been effective in reducing inflammation [14]. The patient may have had a chronic local inflammatory condition in the present case, as adding dexamethasone to the local anesthetic prolonged the effect. Also, we believe that the use of ultrasound helped avoid causing damage to the patellar tendon or periosteum compared to the IFP injection by the conventional blind method.

This is the first report in which the detection of increased brightness on ultrasound of IFP and the injections into the IFP of the patient in question at the initial visit to a pain clinic, triggered an unanticipated additional surgical intervention. It may be worth trying to identify the source of unexplained AKP while being careful to avoid infection and steroid side effects although the clear indications and appropriate content of the drug solution are unknown.

In conclusion, ultrasound evaluation and injection may be beneficial in patients presenting to the pain clinic with AKP and may provide an opportunity for diagnosis.

## Data Availability

Not applicable.

## Abbreviations

AKP: Anterior knee pain
IFP: Infrapatellar fat pad
